# Glycolipids: Linchpins in the Organization and Function of Membrane Microdomains

**DOI:** 10.3389/fcell.2020.589799

**Published:** 2020-10-29

**Authors:** Kei Hanafusa, Tomomi Hotta, Kazuhisa Iwabuchi

**Affiliations:** ^1^Institute for Environmental and Gender-Specific Medicine, Juntendo University Graduate School of Medicine, Urayasu, Japan; ^2^Infection Control Nursing, Juntendo University Graduate School of Health Care and Nursing, Urayasu, Japan

**Keywords:** glycolipid, sphingolipid, membrane microdomain, ceramide synthase 2, phagocytosis, antigen presentation

## Abstract

Membrane microdomains, also called lipid rafts, are areas on membrane enriched in glycolipids, sphingolipids, and cholesterol. Although membrane microdomains are thought to play key roles in many cellular functions, their structures, properties, and biological functions remain obscure. Cellular membranes contain several types of glycoproteins, glycolipids, and other lipids, including cholesterol, glycerophospholipids, and sphingomyelin. Depending on their physicochemical properties, especially the characteristics of their glycolipids, various microdomains form on these cell membranes, providing structural or functional contextures thought to be essential for biological activities. For example, the plasma membranes of human neutrophils are enriched in lactosylceramide (LacCer) and phosphatidylglucoside (PtdGlc), each of which forms different membrane microdomains with different surrounding molecules and is involved in different functions of neutrophils. Specifically, LacCer forms Lyn-coupled lipid microdomains, which mediate neutrophil chemotaxis, phagocytosis, and superoxide generation, whereas PtdGlc-enriched microdomains mediate neutrophil differentiation and spontaneous apoptosis. However, the mechanisms by which these glycolipids form different nano/meso microdomains and mediate their specialized functions remain incompletely understood. This review describes current understanding of the roles of glycolipids and sphingolipids in their enriched contextures on cellular membranes, including their mechanisms of facilitation and regulation of intracellular signaling. This review also introduces new concepts about the roles of glycolipid and sphingolipid-dependent contextures in immunological functions.

## Introduction

Biomembranes consist of a lipid bilayer, containing several types of lipids, in which proteins are embedded. In the fluid mosaic model, transmembrane proteins, such as receptors and binding proteins, are thought to float in a sea of lipids (Singer and Nicolson, 1972). The lipid bilayer is stable and provides a physical boundary that separates the inside from the outside of cells and cellular compartments. The major lipid components of biomembranes are phospholipids, sphingolipids, and cholesterol, all of which have different degrees of lateral motility. The non-homogeneous lateral distribution of these lipid components allows their rearrangement relative to certain membrane-associated proteins, leading to the formation of membrane areas (“microdomains”) with highly differentiated structural and/or functional contextures (Jacobson et al., 1995). Therefore, biomembranes are composed of a variety of distinct small domains that differ in their biophysical characteristics and compositions.

**FIGURE 1:**
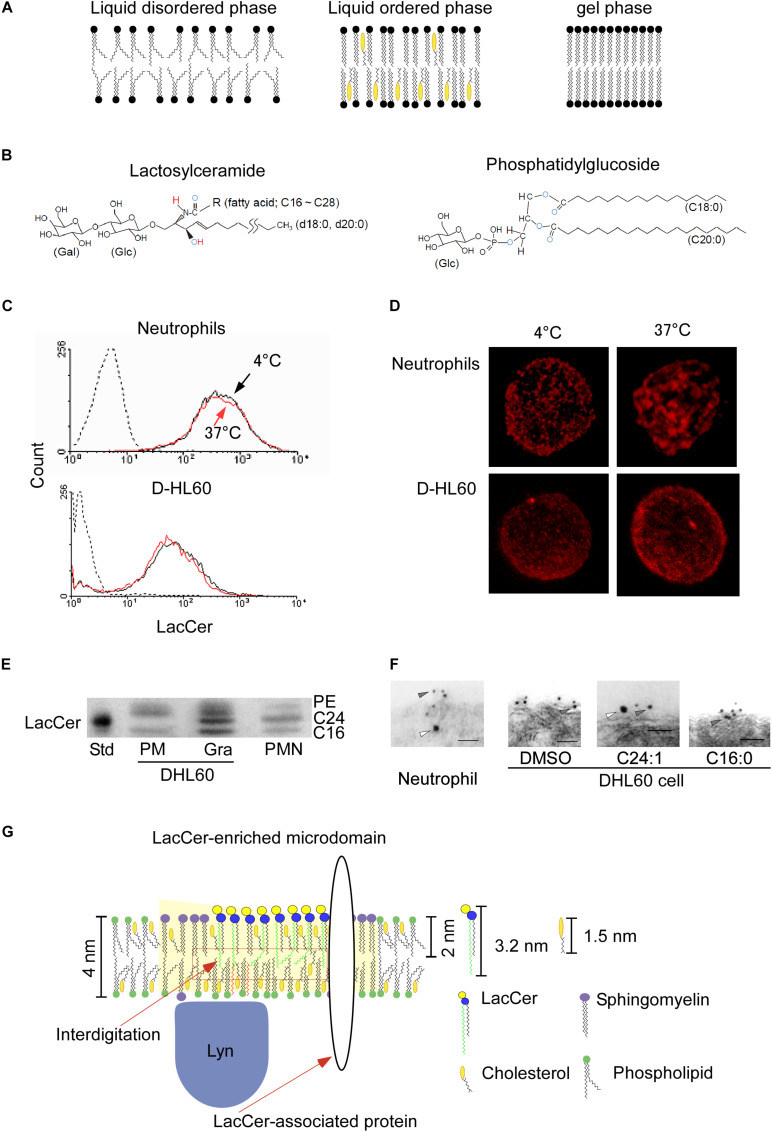
LacCer-enriched microdomain-mediated signaling and functions. **(A)** Typical lipid bilayer phase. **(B)** Structures of lactosylceramide and phosphatidylglucoside. Blue capital, electron acceptor atom. Red capital, electron donor atom. **(C)** Human neutrophils were incubated with Alexa546-anti LacCer monoclonal IgM at 4°C or 37°C for 15 min, and further incubated for 30 min on ice. After fixation, expression of LacCer on their cell surfaces was analyzed by flow cytometry (Iwabuchi et al., 2008). **(D)** The stained cells described in **(C)** were examined by confocal microscope, and 3D images were made by Imaris deconvolution software. **(E)** High performance thin layer chromatography analysis of LacCer in plasma membranes and granular membranes isolated from neutrophils and DHL60 cells (Iwabuchi et al., 2008). The C24-LacCer-containing upper band (C24) was missing from the plasma membranes of DHL60 cells. C16, C16-lacCer containing LacCer. Std, porcine blood-derived C24-LacCer enriched LacCer. PMN, plasma membranes of neutrophils. **(F)** *In situ* association of LacCer with Lyn on plasma membranes of neutrophils or DHL60 cells, as determined by immunoelectron microscopy of ultra thin cryo-sections prepared at ultra low temperature in the absence of organic solvents. Co-localization of LacCer (gray arrowheads) and Lyn (white arrowheads) in plasma membranes of human neutrophils, but not D-HL60 cells (Iwabuchi et al., 2008). Loading of C24:1- but not C16-LacCer onto D-HL60 cells resulted in co-localization of LacCer and Lyn, as in neutrophils. **(G)** Schematic illustration of the structural contexture of Lyn-coupled, LacCer-enriched microdomains. C24-LacCer molecules associated directly with Lyn, which is associated with the inner leaflets of cell membranes via palmitic chains, allowing LacCer-enriched microdomains to mediate neutrophil functions via Lyn. Crosslinking with the photoreactive ^3^H-LacCer analog indicates the presence of several C24-fatty acid chain-containing LacCer-associated proteins, including Lyn and Gαi small G protein (Chiricozzi et al., 2015).

Similar to aqueous solutions, lipids undergo phase transitions in response to certain environmental conditions. Under physiological conditions, membrane bilayers exist in a fluid phase. The primary factor causing phase transitions is the ambient temperature. Above their melting temperature (Tm), individual lipids can move laterally across the surface of the membrane relatively unhindered, a highly fluid state known as the liquid disordered phase (Figure 1A). At temperatures below Tm, however, lipid bilayers exist in a solid-like phase known as the gel phase. Combinations of phospholipids and/or sphingolipids with cholesterol exist in a liquid ordered phase, which possessed both the solid-like qualities of gel phase as well as the high rate of lateral diffusion characteristic of the liquid-disordered phase (Dietrich et al., 2001). Glycosphingolipids (GSLs) are highly enriched on the outer leaflets of plasma membranes. These molecules possess hydroxyl and acetamide groups, which may act as hydrogen bond donors and acceptors, respectively, allowing them to form clusters through *cis* interactions (Figure 1B; Hakomori, 2003). In addition, certain membrane proteins, including glycosylphosphatidylinositol (GPI)-anchored and palmitoylated proteins, which associate with saturated fatty acid chains, tend to cluster in GSL-enriched microdomains (Pike, 2006). Functional liquid-ordered microdomains, also called lipid rafts, have been defined by their GSL- and cholesterol-rich nature, enrichment in GPI-anchored proteins and membrane-anchored signaling molecules, and association with the cytoskeleton (Simons and Ikonen, 1997; Melkonian et al., 1999; Manes et al., 2003; Nagatsuka et al., 2006; Pike, 2006; Kanzawa et al., 2009; Lingwood and Simons, 2010; Nakayama et al., 2018). However, it is difficult to monitor the dynamics of lipid domains formation on biomembranes without affecting the structural contexture of these domains. Thus, the concept of lipid raft-like domains remains obscure (Sezgin et al., 2017; Levental et al., 2020). This review describes the importance of glycolipid- and/or sphingolipid-dependent contextures of biomembranes on the physiological functions of cells.

## How Glycolipids Form Functional Microdomains on Biomembranes

Glycosphingolipids are expressed on the outer leaflets of biomembranes of mammalian cells (Murate et al., 2010, 2015). Carbohydrate moieties of GSLs change sequentially during differentiation (Hakomori, 1981), and the expression patterns of GSL species on plasma membranes differ among cell types (Symington et al., 1985). These findings suggested that each GSL plays specific roles in cell differentiation and functions. Post-translational modifications and/or intermolecular *cis* interactions with surrounding GSLs can induce structural and/or functional changes in membrane proteins. For example, the ganglioside (neuraminic acid-containing GSL) GM3 interacts with EGFR through its neuraminic acid residue (Coskun et al., 2011). GM3 negatively regulates the allosteric structural transition of EGFR from an inactive monomer to an active dimer with signaling properties. In addition, the neutral GSL, lactosylceramide (LacCer), was shown to be involved in glycosylphosphatidylinositol (GPI) side chain galactosylation on endoplasmic reticulum (ER) membranes during the biosynthesis of GPI-anchored proteins (Wang et al., 2020). The lateral heterogeneity of the GSL-enriched microdomains has been detected at nanometer scales (Hakomori, 2003; Iwabuchi et al., 2008; Fujita et al., 2009; Kina et al., 2011). LacCer, GM3 and GM1 have been reported to form microdomains on plasma membranes (Iwabuchi et al., 1998b; Iwabuchi and Nagaoka, 2002; Fratti et al., 2003). Their physicochemical properties differ markedly, with LacCer, GM3 (NeuAcGM3) and GM1 having Tms of 74.4, 35.5, and 19.3°C, respectively. Chain lengths of fatty acyl residues on GSL had a smaller effect on Tm than the complexity of the polar head group (Maggio et al., 1985). Despite having a Tm below body temperature, GM1 forms cholesterol-dependent clusters (liquid ordered domains) on plasma membranes of living cells (Maeda et al., 2007). LacCer- and GM3-enriched microdomains in living cells also contain cholesterol (Iwabuchi et al., 1998a; Iwabuchi and Nagaoka, 2002). GM1 and GM3 differ structurally in only two hexopyranose chains (galactose-β-1-3-N-acetyl galactosamine). Although they contain closely related species of molecular ceramides, GM1 and GM3 form different domains on plasma membranes (Fujita et al., 2007). Treatment with the cholesterol depleting agent methyl-β-cyclodextrin (MBCD) inhibited GM1-dependent influenza A virus endocytosis of human lung epithelial cells (Verma et al., 2018). In contrast, MBCD enhanced LacCer-enriched domain-mediated signal transduction in human neutrophils (Iwabuchi and Nagaoka, 2002). Further studies are necessary to evaluate the roles of carbohydrate moieties of GSLs in the structural and functional contextures of membrane microdomains.

The biochemically isolated membrane microdomains of human neutrophils, also called glycolipid-enriched microdomains (GEM) or detergent-resistant membranes (DRM), contain phosphatidylcholine with several saturated fatty acid chains (Iwabuchi et al., 2008). GPI anchored proteins are also known as microdomain-associated proteins (Pike, 2006). The association between GPI-anchored proteins and membrane microdomains requires the replacement of unsaturated by saturated fatty acid chains, especially the C18:0 chain at sn-2 in the Golgi (Maeda et al., 2007). The lipid moiety of GPI-anchored proteins is glycerophospholipids which have no hydroxyl group, and show only the ability to act as hydrogen bond acceptors (Hakomori, 2003). Phosphatidylglucoside (PtdGlc) is also a member of the glycerophospholipids and forms microdomains on the outer leaflet of plasma membranes, and is composed of C18:0 at sn-1 and C20:0 at sn-2 chains (Murate et al., 2010; Figure 1B). The Tm of PtdGlc is 76.4°C (Takahashi et al., 2012), which is much higher than that of C(18):C(20) phosphatidylcholine (Wang et al., 1995), suggesting that glucosylation of phosphatidic acid dramatically raises the Tm of phospholipids. LacCer and PtdGlc are highly expressed on the outer leaflet of plasma membranes of human neutrophils (Murate et al., 2010, 2015). Therefore, it may be possible that the structural and functional contextures of PtdGlc-enriched microdomains are close to those of LacCer. However, PtdGlc and LacCer form different microdomains than each other on the same living cells, and each mediate different cell functions (Kina et al., 2011). Along with the binding of specific monoclonal antibodies, PtdGlc, but not LacCer, associates with Fas to mediate spontaneous apoptosis in human neutrophils. PtdGlc is preferentially expressed on plasma membranes during the neutrophil differentiation pathway of cord blood CD34(+) cells treated with cytokines (Oka et al., 2009), as well as being involved in neutrophil differentiation itself (Nagatsuka et al., 2003). In contrast, LacCer, which is only expressed on plasma membranes of mature neutrophils, mediates neutrophil chemotaxis, phagocytosis, and superoxide generation (Iwabuchi and Nagaoka, 2002; Sato et al., 2006; Nakayama et al., 2008, 2016). The Src family kinase Lyn is essential for LacCer-mediated neutrophil functions, but is not involved in PtdGlc-mediated apoptosis (Kina et al., 2011). These observations clearly indicate that different types of glycolipid-dependent microdomains are involved structurally and functionally in neutrophil differentiation and immunological functions.

Since LacCer is only expressed on the outer leaflet of plasma membranes (Murate et al., 2015), LacCer cannot migrate to the inner leaflet of the cells by itself. LacCer was found to form large cluster on plasma membranes when human neutrophils were treated with the anti-LacCer antibody T5A7 at 37°C, but not at 4°C, for 15 min, whereas the level of expression of LacCer was not affected by temperature (Iwabuchi et al., 2008; Figures 1C,D). Although neutrophilically differentiated HL-60 cells (D-HL60) cells were also positive for LacCer, LacCer did not form large clusters on the membranes of these cells, and T5A7 did not affect the immunological functions of D-HL60 cells (Iwabuchi and Nagaoka, 2002; Sato et al., 2006; Iwabuchi et al., 2008). These results indicate that crosslinking of LacCer-enriched microdomains, which are connected to the cytoskeleton, can activate neutrophils, resulted in aggregation of their microdomains. In contrast, LacCer-enriched microdomains on D-HL60 cells could not form the required functional contexture.

Ceramide synthase 2 (CerS2) is the enzyme responsible for the synthesis of C22 and C24 ceramides (Laviad et al., 2008; Imgrund et al., 2009; Pewzner-Jung et al., 2010a). Elongation of very long chain fatty acids protein 1 (ELOVL1) is also essential for the production of C24 fatty acid-containing SLs. ELOVL1 siRNA and CerS2 siRNA reduced Lyn activation in HeLa cells (Ohno et al., 2010). In microsomal lipid extract obtained from CerS2 null mice, biophysical properties of biomembrane showed higher fluidity and morphological changes (Pewzner-Jung et al., 2010b). The major molecular species of LacCer in human neutrophil plasma membranes include C16:0, C24:0, C24:1, and C22:0, whereas the molecular species of LacCer in plasma membranes of D-HL60 cells contain little C24:0 and C24:1 (Figure 1E). Reconstitution experiments showed that introducing C24-LacCer into plasma membranes of D-HL60 cells resulted in the formation of Lyn-associated, LacCer-enriched microdomains (Figure 1F) and that treatment of these cells with T5A7 induced immunological functions. Moreover, cross-linking using azide-photoactivatable ^3^H-LacCer showed that C24:0-LacCer, but not C16:0-LacCer, was directly associated with Lyn in living cells (Chiricozzi et al., 2015). These findings suggest that C24 fatty acid chains of GSLs are essential for the GSL-mediated *trans-*bilayer signaling systems of GSL-enriched microdomains.

The cholesterol in membrane microdomains causes spaces in the central part of the membrane to be unoccupied, allowing the interdigitation of longer alkyl chains (Kusumi et al., 2004). The thickness of plasma membranes has been estimated to be about 4 nm (Teneberg et al., 2004; Kain et al., 2014; Figure 1G). In comparison, the fatty acid chain lengths of C24:0- and C24:1-LacCer species are about 3.2 nm, more than 40% the half hydrophobic thickness of biomembranes (Tanford, 1980). Nuclear magnetic resonance (NMR) of artificial lipid bilayers has indicated that C24, but not C22:0 and shorter, fatty acid chains of LacCer interdigitate with fatty acids of the opposing monolayer (Grant et al., 1987). Atomistic molecular dynamics simulations have indicated that very long fatty acid chain-containing GM1 was connected to the inner leaflet of membranes via interdigitation (Manna et al., 2017). LPS treatment of hepatocytes from CerS2 null mice did not induce internalization of tumor necrosis factor receptor 1 (TNFR1) or TNFR1-mediated cell death, nor did it affect Fas-dependent apoptosis (Ali et al., 2013). The loss of C24 fatty acid chain-containing sphingolipids (C24-SLs) directly affected insulin receptor translocation and subsequent signaling in mouse liver cells (Park et al., 2013). Translocation of granulocyte-colony stimulating factor receptor (GCSFR) into membrane microdomains and subsequent Lyn activation were not observed in bone marrow cells from CerS2 null mice (Kurz et al., 2019). These observations using CerS2 null mice strongly indicated that C24 fatty acid chains of GSLs and/or sphingomyelin play essential roles in several biological activities of these cells.

## Glycolipid-Enriched Membrane Microdomains Change Their Contextures During Their-Dependent Cellular Functions

Mammalian cells express several kinds of glycolipids, which mediate glycolipid-specific cell functions (Chiricozzi et al., 2015; Furukawa et al., 2018; Groux-Degroote et al., 2018; Iwabuchi, 2018; Nakayama et al., 2018). LacCer is highly expressed on the plasma membranes of human phagocytes, especially neutrophils. Among GSLs, only LacCer binds to a wide range of infectious microorganisms in humans, including *Mycobacterium tuberculosis* (*M. tuberculosis*), *Shigella dysenteriae, Vibrio cholerae, E. coli, Candida albicans*, *Bordetella pertussis*, and *Helicobacter pylori* (Karlsson, 1989; Saukkonen et al., 1992; Angstrom et al., 1998; Sato et al., 2006; Nakayama et al., 2016). LacCer-enriched microdomains specifically bind to β-1,6 long glucopyranose side chains branched with a β-1,3 glucopyranose of β-glucan expressed on *Candida albicans* (Sato et al., 2006). Lipoarabinomannan (LAM), which is commonly expressed on mycobacteria, has a mannan core structure, consisting of a 21–34 residue α(1→6)-mannopyranose backbone and five to 10 α(1→2)-mannopyranose side chains (Mishra et al., 2011). Regardless of pathogenicity, LacCer-enriched microdomains that react with *Mycobacteria* form through binding to the mannan core structure (Nakayama et al., 2016). The phosphatidylinositol-capped LAM (PILAM) α1,2-mannosyltransferase deletion mutant (Δ*MSMEG_4247*), which lacks the α1,2-monomannose side branches of the LAM mannan core, did not bind to LacCer or induce phagocytosis, indicating that the mannan core structure is essential for phagocytosis by human phagocytes. These findings suggest that LacCer-enriched microdomains on human neutrophils act as pattern recognition receptors (PRRs) against microorganisms through the common structural patterns of fungal β-glucans and mycobacterial LAMs. Interestingly, mouse neutrophils expressed low levels of LacCer, and LacCer-mediated functions were limited (Iwabuchi et al., 2015). Taken together, these findings indicated that LacCer-enriched microdomains on human cells are specifically responsible for the immunological activities of these cells against pathogenic microorganisms.

In human neutrophils, 90% of LacCer is contained within microdomains on intracellular biomembranes and does not translocate to plasma membranes (Iwabuchi and Nagaoka, 2002; Iwabuchi et al., 2008). Several types of cellular granules sequentially fuse to bacteria-containing phagosomes. Membrane contact sites (MCSs) are present at the fusion sites of organelles and phagosomes, especially at the fusion sites of the ER with endolysosomes, phagosomes, the Golgi and mitochondria (Yamaji and Hanada, 2015; Hariri et al., 2016; Nunes-Hasler and Demaurex, 2017; Hanada, 2018; Tamura et al., 2019; Tu et al., 2020). In each MCS, membrane microdomains on organelles form sub-regions (Nunes-Hasler and Demaurex, 2017). However, the types of molecules essential to form functional contextures of MCSs are still not completely known. Phagosome maturation in the mouse macrophage cell line RAW264.7 cells was associated with a decrease in CerS2, but an increase in C24-ceramide molecules (Pathak et al., 2018).

Granular LacCer-enriched microdomains reorganize into microdomains on phagosomal membranes (Nakayama et al., 2016). Lyn-coupled LacCer-enriched microdomains participate in the phagocytosis of mycobacteria (Nakayama et al., 2016), whereas LacCer-enriched microdomains coupled to the Src family kinase Hck form a functional contexture for phagolysosome maturation. Importantly, Hck was not associated with LacCer-enriched microdomains in resting neutrophils, indicating that Hck association with LacCer-enriched microdomains is indispensable for fusion of lysosomes to phagosomes. Intracellular pathogens, such as *M*. *tuberculosis*, target membrane microdomains of target cells (Manes et al., 2003). Pathogenic mycobacteria express ManLAM (Kaur et al., 2008), which inhibits the fusion of lysosomes to phagosomes, enabling these bacteria to survive in host phagocytes (Diacovich and Gorvel, 2010). Pathogenic mycobacteria disrupt the coupling of LacCer-enriched microdomains with Hck on phagosomal membranes through the mannose-capping motif of ManLAM (Nakayama et al., 2016). Collectively, to kill microorganisms, human neutrophils must express Lyn-coupled LacCer-enriched microdomains on plasma membranes, trapping the microorganisms. Phagolysosome maturation requires LacCer-enriched microdomains to be reconstructed with Hck on phagosomal membranes, thereby altering their functional contexture. The biomembranes of human phagocytes contain several different LacCer-enriched microdomains, forming appropriate functional contextures for their specialized functions.

## Role of Sphingolipids and Their Metabolites on Antigen Presentation

**FIGURE 2:**
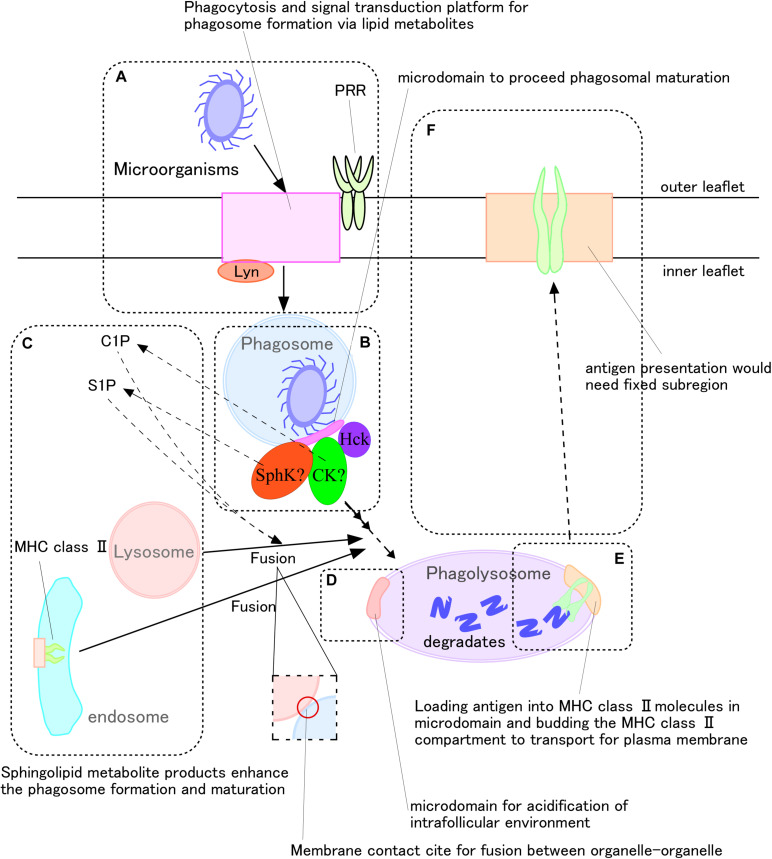
Very long fatty acid chain-containing sphingolipid metabolites mediate antigen presentation during phagocytosis of microorganisms. **(A,B)** Recognition of microorganisms generates sphingomyelin metabolites, including ceramide, as well as their conversion to ceramide-1-phosphate and sphingosine-1-phosphate. **(C)** These metabolites promote phagolysosome formation. **(D)** V-ATPase promotes phagosome maturation by, for example, fusing with lysosomes to degrade microorganisms, and reduced pH adjusts MHC class II activity. **(E,F)** Following antigen loading onto MHC class II molecules, the complexes translocate to the plasma membrane for antigen presentation.

Microdomains have been suggested to be essential for the antigen presentation process against microorganisms (Poloso et al., 2004; Shrestha et al., 2014; Zuidscherwoude et al., 2014). Antigen presentation by macrophages and dendritic cells begins with these cells engulfing microorganisms. The phagosomes that form within these cells fuse with lysosomes, yielding phagolysosome, which digest the engulfed microorganisms (Figure 2). These degradation products in phagolysosomes bind to molecules of the major histocompatibility complex (MHC) (Figure 2D). Finally, these antigen-MHC molecular complexes are transported to the cell surface (Figure 2F). Assessment of RAW264.7 showed that CerS2 accumulated in phagosomes during engulfment, and that C24-ceramides were enriched in late phagolysosomes (Pathak et al., 2018).

Sphingomyelin and its metabolites are important during the recognition phase of antigen presentation. Knock-out of the gene encoding sphingomyelin synthase (SMase) 1 in human monocytic leukemia cell line U937 cells was found to inhibit the phagocytosis of *M. tuberculosis.* Moreover, knockout of the gene encoding serine palmitoyltransferase 2 impaired clustering of PRRs, such as Dectin-1, in RAW264.7 cells (Niekamp et al., 2019). The phosphatase CD45 did remained at the receptor-pathogen contact site of nascent phagosomes. In general, SM, one of the main components of membrane microdomains, is distributed uniformly on the outer leaflets of plasma membranes of cells, including human neutrophils (Zachowski, 1993; Murate et al., 2015). Recently, our group found SM clusters on the inner leaflets of plasma membranes of human neutrophils (Murate et al., 2015). Sphingomyelinase, ceramidase, and sphingosine kinase, along with other related enzymes, are present in the cell cytosol (Milhas et al., 2010). After recognition of antigen, intracellular Src and Syk are phosphorylated (Edberg and Kimberly, 1994), and changes in intracellular Ca^2+^ concentrations during phagolysosome formation activate phospholipase (PL)C or D (Sawyer et al., 1985; Corrotte et al., 2006). Higher concentrations of intracellular Ca^2+^ activate ceramide kinase to generate C1P (Mitsutake and Igarashi, 2005), which enhances phagolysosome maturation (Hinkovska-Galcheva et al., 2005). S1P has also been found to mediate Ca^2+^-dependent phagolysosome maturation (Malik et al., 2003; Figure 2C). To produce S1P, ERK1/2 phosphorylates sphingosine kinase (SphK) at Ser255, and the phosphorylated SphK translocates to the plasma and phagosome membranes during phagocytosis (Thompson et al., 2005). Intracellular S1P lyase 2, which can digest S1P, inhibits phagolysosome formation in macrophages (Abu Khweek et al., 2016), whereas C1P regulates the activity of SphK (Nishino et al., 2019). *M*. *tuberculosis* can avoid destruction by macrophages via inhibition of SphK activity (Malik et al., 2003). Therefore, cytosolic leaflet-associated SM would constitute a starting material for microdomains on phagosomal membranes, promoting phagolysosome maturation and the antigen presentation process.

## Author Contributions

KH searched the references, wrote the manuscript, and made a figure. TH searched the references and wrote the manuscript. KI organized the gathered references, made the concept, wrote the manuscript, and made a figure. All authors contributed to the article and approved the submitted version.

## Conflict of Interest

The authors declare that the research was conducted in the absence of any commercial or financial relationships that could be construed as a potential conflict of interest.
